# Telehealth among Medicare Advantage beneficiaries with and without Alzheimer's Disease and Related Dementias (ADRD), 2018–2024: from pandemic-era surge to post-pandemic decline

**DOI:** 10.1186/s12913-026-14174-1

**Published:** 2026-03-13

**Authors:** Hailemichael B. Shone, Sih-Ting Cai, Kosali Simon, Jennifer L. Carnahan, Malaz Boustani

**Affiliations:** 1 The Paul H. O’Neill School of Public and Environmental Affairs, Indiana University, Bloomington, IN USA; 2 Irsay Institute, Indiana University, Bloomington, IN USA; 3 Center for Health Innovation and Implementation Science, School of Medicine, Indiana University, Indianapolis, IN USA; 4 Center for Aging Research, Regenstrief Institute, Indianapolis, IN USA; 5 National Bureau of Economic Research, Cambridge, MA USA

**Keywords:** Telehealth, Medicare Advantage, Alzheimer's disease and related dementias

## Abstract

**Background:**

Telehealth has become a vital element of healthcare delivery, especially among Medicare Advantage (MA) beneficiaries. Yet its use among individuals with Alzheimer’s Disease and Related Dementias (ADRD) – a population with complex biopsychosocial needs – remains understudied.

**Methods:**

We conducted an observational cohort study of MA beneficiaries aged 65 and older using Optum’s de-identified Clinformatics® Data Mart Database (January 2017–June 2024). A matched sample of enrollees with and without ADRD was constructed. Difference-in-differences and event study analyses were applied to evaluate adjusted differences in quarterly telehealth utilization before and after the 2020 Medicare telehealth policy expansion during the COVID-19 public health emergency. The primary outcome was a quarterly indicator of any telehealth use, defined among enrollee-quarters with any medical claim.

**Results:**

Among 421,131 MA beneficiaries, telehealth use increased sharply in Q2 2020 for both ADRD and non-ADRD groups, peaking at 33.9 and 30.6% respectively. Use declined and stabilized by 2021, remaining slightly higher among beneficiaries with ADRD by Q2 2024. Adjusted DiD analyses showed a small but statistically significant post-2020 increase in telehealth use for the ADRD group compared to the non-ADRD group (2.58 percentage points; p < 0.01), with the gap narrowing over time.

**Limitations:**

Findings are based on one national MA claims database and may not generalize to other populations or settings.

**Conclusion:**

MA beneficiaries with and without ADRD rapidly adopted telehealth following the 2020 policy expansion. Although use was initially higher among those living with ADRD, utilization converged over time. As telehealth policy evolves, it is critical to address barriers for cognitively impaired populations.

**Supplementary Information:**

The online version contains supplementary material available at 10.1186/s12913-026-14174-1.

## Background

Over the past five years, telehealth has transitioned from a temporary measure during the COVID-19 pandemic into an increasingly central component of the U.S. healthcare delivery system. Pre pandemic, a 2018 congressional policy authorized Medicare Advantage (MA) plans to include telehealth services as part of their basic benefit packages – rather than only a supplemental benefit – starting in 2020 [1, 2]. When the COVID-19 public health emergency (PHE) began in early 2020, the Centers for Medicare & Medicaid Services (CMS) implemented emergency policies that dramatically expanded telehealth reimbursement and access under both Fee For Service (FFS) Medicare and MA.

Initially intended to reduce in-person contact, telehealth quickly became a vital tool for delivering a wide range of services – including routine care, behavioral health, and chronic disease management [3]. For older adults with chronic conditions, telehealth helped address longstanding barriers to care, including mobility limitations, transportation challenges, immunocompromise, and geographic isolation [4–6]. Although the PHE officially ended in May 2023, many of the expanded telehealth flexibilities were extended as of this writing, to be re-assessed in the near future [7, 8].

One population that may particularly benefit from telehealth is individuals living with Alzheimer’s Disease and Related Dementias (ADRD), progressive neurodegenerative conditions that impair an individual’s memory, cognition, communication, and the ability to manage daily tasks. Older adults with ADRD often require ongoing medical care, frequent monitoring, and substantial caregiver support, which can make accessing in-person services especially difficult for both patients and their caregivers. Telehealth may ease these barriers through several mechanisms. First, it can reduce transportation and mobility burdens that are common in ADRD, especially when patients require supervision or assistance to attend clinic visits [9]. Second, telehealth care can facilitate timely follow-up for medication adjustments and monitoring behavioral symptoms [10]. Third, it can support caregiver involvement, which allows caregivers to participate and may improve continuity of care [11]. However, telehealth may not uniformly reduce barriers for this population. For those with cognitive and sensory impairment, technology access and literacy constraints, and limited caregiver support, telehealth could be challenging. As the U.S. population ages, the prevalence of ADRD is growing rapidly: an estimated 6.7 million Americans aged 65 and older are currently living with ADRD, a number projected to double by 2060. In 2023, the total national cost of care for individuals with ADRD was estimated at $345 billion, placing immense strain on families, caregivers, and the healthcare system [12].

The Medicare Advantage program has become the dominant model in Medicare, enrolling over half of all Medicare beneficiaries as of 2023 [13]. MA plans operate under a capitated, managed care model and some plans offer greater flexibility than FFS. Prior to the 2020 telehealth policy expansion, many MA plans offered telehealth access as part of their benefit packages [2]. In contrast, FFS Medicare telehealth coverage was limited largely to beneficiaries living in rural areas and required patients to travel to designated clinical sites for telehealth visits [14]. While rates of telehealth use in FFS Medicare for the pre-pandemic period are shown to be extremely low [15], there is very little published research on telehealth use in MA. As MA continues to shape how care is delivered for a growing share of older adults – including those with ADRD – it is increasingly important to understand how telehealth is being used among MA enrollees, and whether this mode of care delivery is meeting the needs of high-need, medically complex populations.

To our knowledge, few published studies have examined telehealth use specifically among individuals with ADRD, and those that do are limited by small samples, narrow geographic focus, or short observation windows. For example, Wang et al. [16]. used data from the Medicare Current Beneficiary Survey (MCBS) and found that, despite greater pre-pandemic telehealth access, MA enrollees did not use it more than FFS enrollees during the pandemic. Another study in two integrated delivery systems reported sustained telemedicine use post-COVID among those with ADRD, but only in primary care and without comparison to those without ADRD [17]. Other studies have shown higher overall healthcare utilization in MA vs. FFS overall [18], as well as for populations with ADRD diagnosis [19]. While MA enrollees had greater access to telehealth services than those in FFS, use was generally low in both groups [20]. However, these studies did not specifically examine telehealth utilization patterns for individuals with ADRD. This leaves a critical gap in understanding how MA enrollees with ADRD have engaged with telehealth – particularly over time, and in the context of rapid policy and technological change.

To address gaps in the telehealth literature, we examine telehealth utilization among MA beneficiaries with and without ADRD across three key phases: before, during, and after the 2020 Medicare telehealth expansion. “Before” captures pre-expansion baseline use under more restrictive Medicare telehealth rules; “during” reflects the immediate post-expansion period when policy flexibilities and pandemic-related disruptions rapidly shifted care delivery toward remote modalities; and “after” represents the subsequent period when in-person care partially rebounded but many Medicare telehealth flexibilities and provider/patient telehealth habits persisted. This longitudinal approach enables us to describe how telehealth use evolved over time within the MA population and whether changes differed for beneficiaries with ADRD relative to those without. We hypothesized that telehealth use would rise sharply after the 2020 expansion and remain above pre-2020 level for both groups, and the increase would be larger among beneficiaries with ADRD.

## Methods

### Data source and study population

The study uses Optum’s de-identified Clinformatics^®^ Data Mart Database (Optum^®^ CDM or Optum Clinformatics^®^), which contains administrative health claims from individuals enrolled in large commercial and MA health plans. Optum Clinformatics^®^ includes detailed patient-level information on enrollment, medical and pharmacy claims, health care costs, and utilization.

**Fig. 1:**
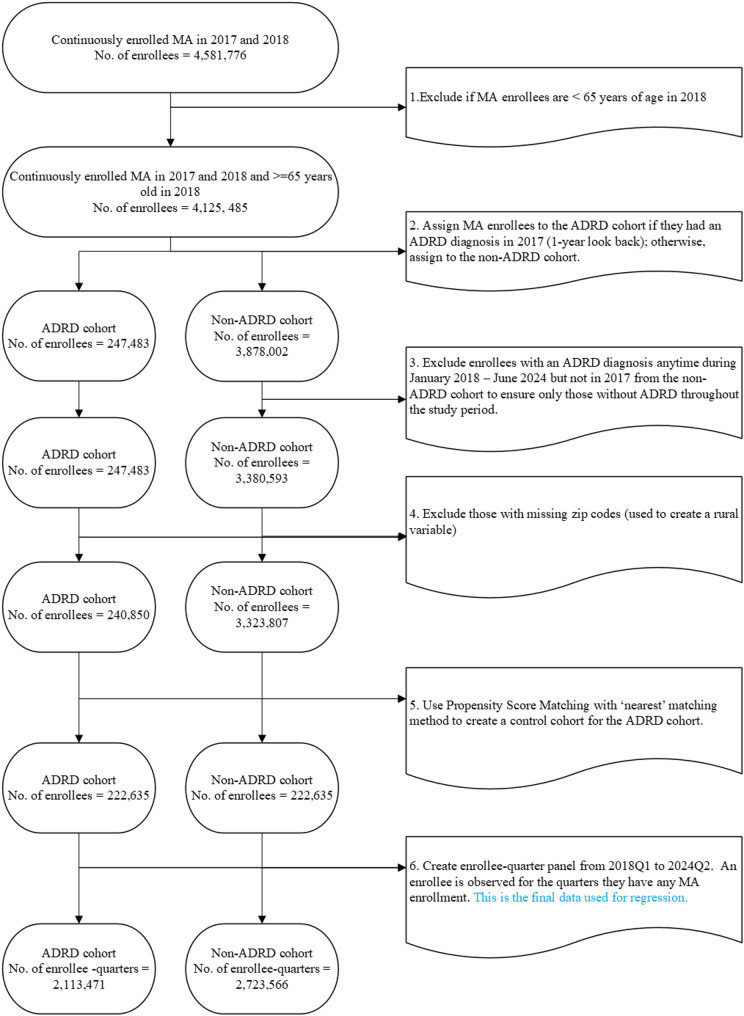
Process of sample construction

We created cohorts of MA enrollees who were continuously observed in MA plans in both 2017 and 2018 and were aged 65 years or older as of 2018. All individuals included in this analysis were observed in 2017 and for at least one subsequent year (2018) through 2024, and were followed until the end of data availability or until they were no longer observed in enrollment (e.g., disenrollment or death). We then identified individuals with ADRD using ICD-10 diagnosis codes used in the Bynum/Grodstein claims-based algorithm [21]. Enrollees were classified as having ADRD if they had at least one medical claim containing an ADRD related ICD-10 diagnosis code during a one-year lookback period in 2017, including Alzheimer’s disease (G30.*) and related dementias (F01.*, F02.*, F03.*, and G31.*).We have included the list of the ICD 10 codes in the Supplemental Methods. Based on the ADRD status, we separated enrollees into two mutually exclusive groups: the ADRD group (the treated cohort) included individuals who presented with an ADRD diagnosis in the lookback period of 2017, and the non-ADRD group (the comparison cohort) included those without an ADRD diagnosis in 2017 and at any point observed from 2018 to 2024. We excluded those who were present in 2017 with no ADRD diagnosis but received a subsequent diagnosis afterward during our study period, resulting in an analytical sample with consistent ADRD classification over time. Figure 1 provides a detailed description of the sample construction process.

### Main outcome measure

Our main outcome variable is an indicator of whether an enrollee used any telehealth services in a quarter during the study period. To identify the use of telehealth for each enrollee, we searched for the use of any of the following CPT modifiers 93, 95, GT, GQ, FQ or place of service (POS) codes 02 or 10 [7, 22].

### Analysis approach

Because MA enrollees with ADRD differ substantially from those without ADRD in terms of age, sex, and underlying health conditions, we employed Propensity Score Matching (PSM) to construct a comparison cohort with similar baseline characteristics prior to difference-in-differences analysis (DiD) analysis. PSM improves comparability between ADRD and non-ADRD enrollees by balancing observable characteristics and reducing confounding due to systematic differences in demographics and health status [23].

Using logistic regression with demographic characteristics and comorbidities measured in 2017, we estimated propensity scores and matched ADRD and non-ADRD enrollees using 1:1 nearest-neighbor matching without replacement and a caliper of 0.2. Matching variables included age in years, sex, dual Medicare and Medicaid eligibility, rural residence, and baseline indicators for comorbid conditions. Rural residence was defined using the 2013 National Center for Health Statistics (NCHS) Urban–Rural Classification Scheme for Counties, with non-core counties categorized as rural [24], while dual-eligibility status was included to account for socioeconomic and coverage differences that may influence telehealth access. Comorbidities were selected a priori based on prior work describing common chronic disease burden among individuals with ADRD [25], including asthma, chronic obstructive pulmonary disease, diabetes, hypertension, cancer, arthritis, congestive heart failure, coronary artery disease, stroke, kidney disease, liver disease, and depression. This matching procedure improved covariate balance and strengthened the internal validity of subsequent difference-in-differences estimates.

Using the matched sample, we estimated pooled ordinary least squares linear probability models in a difference-in-differences (DiD) framework (Supplemental Methods) to examine differential changes in telehealth use among MA beneficiaries with and without ADRD following the 2020 Medicare telehealth policy expansion, relative to the pre-expansion period. Models included indicators for ADRD status, the post-expansion period beginning in 2020 quarter 1, and their interaction, with standard errors clustered at the enrollee level. We excluded enrollee-quarters with no medical claims because telehealth use is conditional on seeking care, and inclusion of such quarters could mechanically inflate the share of non-users.

To assess the parallel trends assumption underlying the DiD design, namely that trends in telehealth use would have evolved similarly for beneficiaries with and without ADRD in the absence of the policy expansion, we estimated an event-study specification. This model includes interactions between ADRD status and quarter indicators, allowing relationship between calendar time and telehealth use to vary flexibly between groups over time rather than imposing a common time pattern. The resulting pre-2020 interaction coefficients provide a diagnostic for differential pre-trends prior to 2020. Analyses were conducted using R (version 4.3) and the fixest package for estimation.

## Results

### Descriptive results

**Fig. 2:**
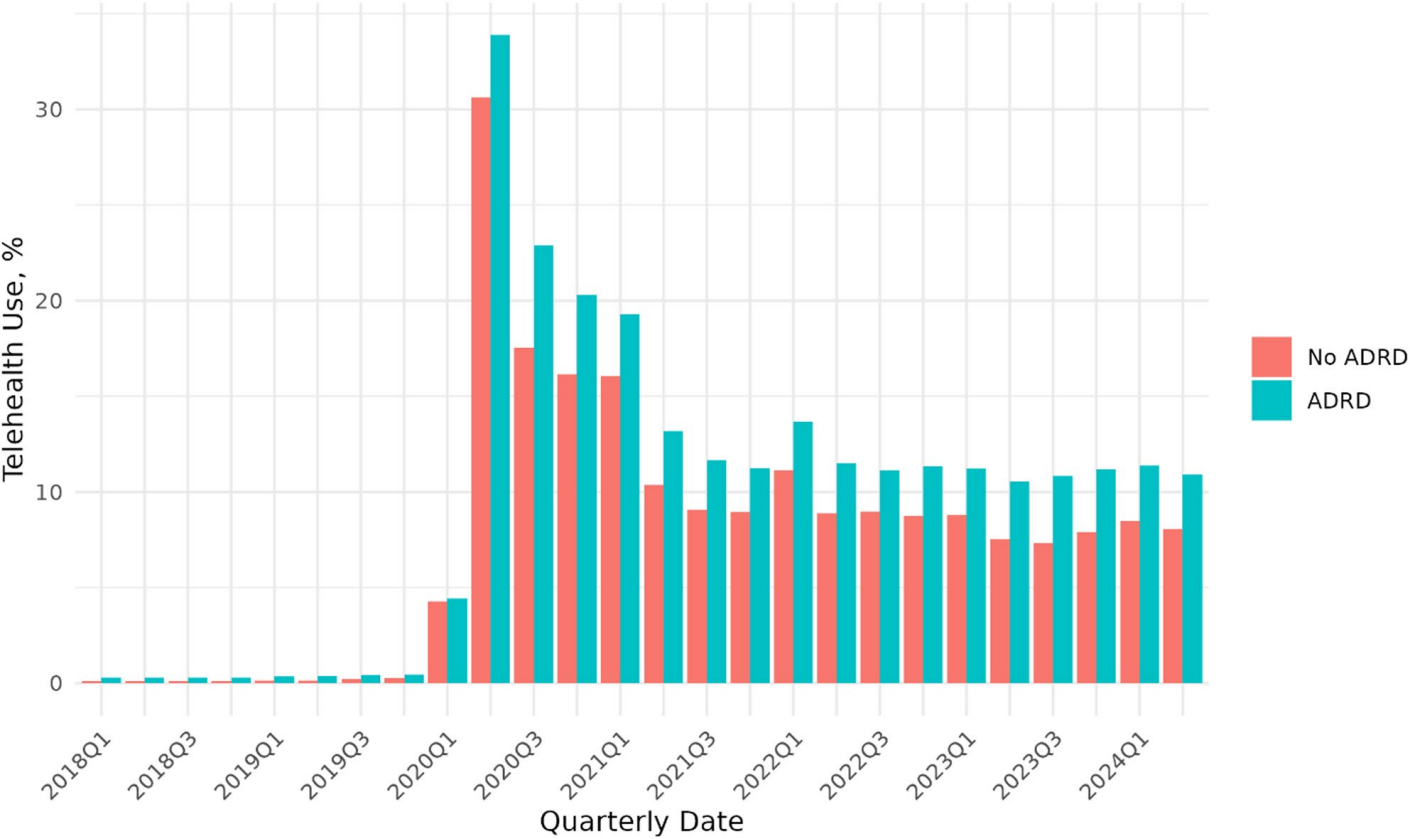
Trend in telehealth use (% of beneficiaries with any telehealth use) by ADRD status among MA Enrollees, 2018–2024. Notes: Values represent the quarterly percentage of Medicare Advantage beneficiaries with any telehealth use (≥ 1 telehealth visit) in that quarter, stratified by ADRD status. Estimates are calculated in the propensity score–matched sample at the beneficiary–quarter level and are restricted to enrollee-quarters with ≥ 1 medical claim. Prior to 2020Q1, telehealth use was low in both groups (< 1%), but consistently higher among beneficiaries with ADRD than among those without ADRD

Figure 2 presents quarterly trends in the use of telehealth by the ADRD vs. non-ADRD group in the matched data. Although the proportion of enrollees with telehealth use was low among both groups before the 2020 policy expansion (less than 1%), telehealth use was consistently higher among individuals living with ADRD compared to those without ADRD. Both groups experienced a significant increase following the 2020 policy expansion (peaking at 30.6% for the non-ADRD group and 33.9% for the ADRD group, in Q2 2020), although telehealth utilization declined slightly among both groups. At the end of our study period (2024 Q2), about 8% and 11% of enrollees without and with ADRD diagnoses used telehealth respectively.

Table 1 presents the baseline and post-matching characteristics among MA enrollees with and without ADRD diagnoses in the pre-telehealth policy expansion period. Our analytical sample included 3,564,657 MA beneficiaries observed during the pre-expansion period. At the baseline, among MA enrollees without ADRD, the mean (SD) age was 73.5 (6.2) years. Females accounted for 1,897,462 individuals (57.1%), 302,233 enrollees (9.1%) were dually eligible, and 162,733 (4.9%) resided in rural areas. In contrast, among enrollees with ADRD, the mean (SD) age was 81.1 (6.2) years; 157,275 (65.3%) were female, 80,685 (33.5%) were dually eligible, and 11,079 (4.6%) lived in rural areas. Compared to those with normal cognition, MA enrollees living with ADRD were older, more likely to be female, highly likely to be dually eligible, and had a higher prevalence of chronic conditions. To account for these substantial baseline differences, we applied PSM to construct a comparable sample of ADRD and non-ADRD enrollees for subsequent analyses. Table 1 and Figure A1 illustrate the standardized mean differences before and after matching, demonstrating improved covariate balance across key characteristics.

**Table 1:** Covariate balance: baseline (2017) sample characteristics before and after propensity score matching

Variable	Before matching				After matching		
	ADRD	Non- ADRD	Std. mean diff.		ADRD	Non- ADRD	Std. mean diff.
Age, years	81.1	73.5	1.236		80.7	80.6	0.013
Female, %	65.3	57.1	0.17		63.9	64.1	0.005
Dual eligibility	33.5	9.1	0.625		29.6	28.4	0.027
Rural residence	4.6	4.9	0.026		4.6	4.4	0.007
Asthma, %	6.7	6.1	0.026		7.0	7.1	0.003
COPD, %	20.0	10.2	0.278		20.2	21.1	0.02
Diabetes, %	36.9	26.6	0.223		36.7	37.2	0.01
Hypertension, %	81.9	60.5	0.486		80.8	82.2	0.035
Cancer, %	15.5	14.1	0.039		16.1	17.0	0.023
Arthritis	38.4	23.4	0.327		37.3	37.7	0.007
Congestive Heart Failure	23.0	7.2	0.454		22.1	23.2	0.025
Coronary Artery Disease	31.1	16.3	0.353		30.8	31.6	0.017
Stroke	13.8	2.3	0.429		12.7	12.3	0.012
Kidney Disease	35.2	14.5	0.493		34.3	35.6	0.029
Liver Disease	4.7	4.2	0.027		4.9	5.0	0.002
Depression	37.0	10.2	0.665		33.1	33.1	0.002
Enrollees (N)	240,850	3,323,807			222,635	222,635	

Note: Columns 1 and 2 displays the 2017 characteristics of beneficiaries who were enrolled in 2017, and either had a diagnosis of ADRD in 2017 and throughout their inclusion in the data (Column 1), or, had no ADRD diagnosis in 2017 and remained without an ADRD diagnosis for their entire observation period in the data since then (Column 2). Beneficiaries who did not have an ADRD diagnosis in 2017 but obtained a diagnosis since then were excluded from the sample. In Columns 4 and 5, propensity scores were used to match participants based on baseline covariates. Balance was assessed using standardized mean differences; values < 0.1 indicate acceptable balance. Characteristics are presented before and after matching to show covariate balance

### Comparison of telehealth use by ADRD vs. non-ADRD longitudinal cohorts

Although Fig. 2 displays descriptive statistics, these trends do not provide statistical tests of differences between groups or over time. The DiD results enable statistical implications to be tested. Table 2 presents the results from the DiD analysis estimating the change in telehealth use among MA beneficiaries before and after the 2020 Medicare telehealth policy expansion, comparing individuals with and without ADRD. In both unadjusted and covariate-adjusted models, telehealth use increased significantly in the post period for both non-ADRD and ADRD MA enrollees. Before COVID-19, the use of telehealth services was approximately 0.34 percentage points (95% CI, 0.29, 0.40) higher among MA beneficiaries with ADRD compared to those without. Following the telehealth policy expansion during the COVID period, there was an overall increase in telehealth use for both groups compared to the pre-COVID period. For MA beneficiaries without ADRD, telehealth use increased by about 5.04% points after the telehealth policy expansion (95% CI, 4.94, 5.14). For MA beneficiaries with ADRD, the increase was higher – increasing by 7.62 percentage points. This corresponds to an increase of approximately 51% relative to the pre-COVID telehealth use rate among beneficiaries with ADRD. Thus, compared to MA beneficiaries with no ADRD diagnosis, the post-policy increase in telehealth use among MA beneficiaries with ADRD is higher by about 2.58 percentage points (95% CI, 2.40, 2.77).

**Table 2:** Difference-in-Differences estimation: telehealth use among MA beneficiaries with and without ADRD, after and before the 2020 Medicare telehealth policy expansion

Variable	Unadjusted Model (Estimate, 95% CI)	Adjusted Model (Estimate, 95% CI)
Constant	4.38 [4.34, 4.42]	14.70 [14.10, 15.31]
ADRD	0.29 [0.23, 0.34]	0.34 [ 0.29, 0.40]
Post	5.21 [5.11, 5.31]	5.04 [ 4.94, 5.14]
ADRD x Post	2.69 [2.51, 2.87]	2.58 [ 2.40, 2.77]
Demographics		
Age		-0.16 [-0.17, -0.15]
Female		0.62 [ 0.53, 0.70]
Dual Eligibility		0.45 [ 0.36, 0.55]
Rural Residence		-0.69 [-0.88, -0.50]
Comorbidities		
Asthma		1.29 [ 1.11, 1.48]
COPD		0.20 [ 0.09, 0.32]
Diabetes		0.49 [ 0.40, 0.58]
Hypertension		0.48 [ 0.37, 0.59]
Cancer		0.51 [ 0.40, 0.62]
Arthritis		0.71 [ 0.63, 0.80]
Congestive Heart Failure		0.24 [ 0.13, 0.35]
Coronary Artery Disease		0.49 [ 0.40, 0.59]
Stroke		0.10 [-0.03, 0.23]
Kidney Disease		0.15 [ 0.06, 0.24]
Liver Disease		0.50 [ 0.30, 0.71]
Depression		1.39 [ 1.30, 1.49]
Enrollee-quarter (N)	4,837,037	4,837,037
Unique enrollees	421,131	421,131

Notes: Estimates are from pooled OLS linear probability model estimated using a difference-in-differences (DID) design. The unit of analysis is the enrollee-quarter, and the outcome is an indicator for any telehealth use during the quarter. *Post* indicates the post-2020 period. ADRD indicates whether the enrollee was diagnosed with Alzheimer’s disease or related dementias. The interaction term (*ADRD × Post*) represents the DID estimate, capturing the differential change in telehealth use among enrollees with ADRD in the post period relative to those without ADRD. Standard errors are clustered at the enrollee (patient) level. All coefficients and 95% confidence intervals are reported in percentage points. The adjusted model additionally controls for **baseline** age (years), sex, comorbid conditions (asthma, COPD, diabetes, hypertension, cancer, arthritis, congestive heart failure, coronary artery disease, stroke, kidney disease, liver disease, depression), dual eligibility status, and rural residence

**Fig. 3:**
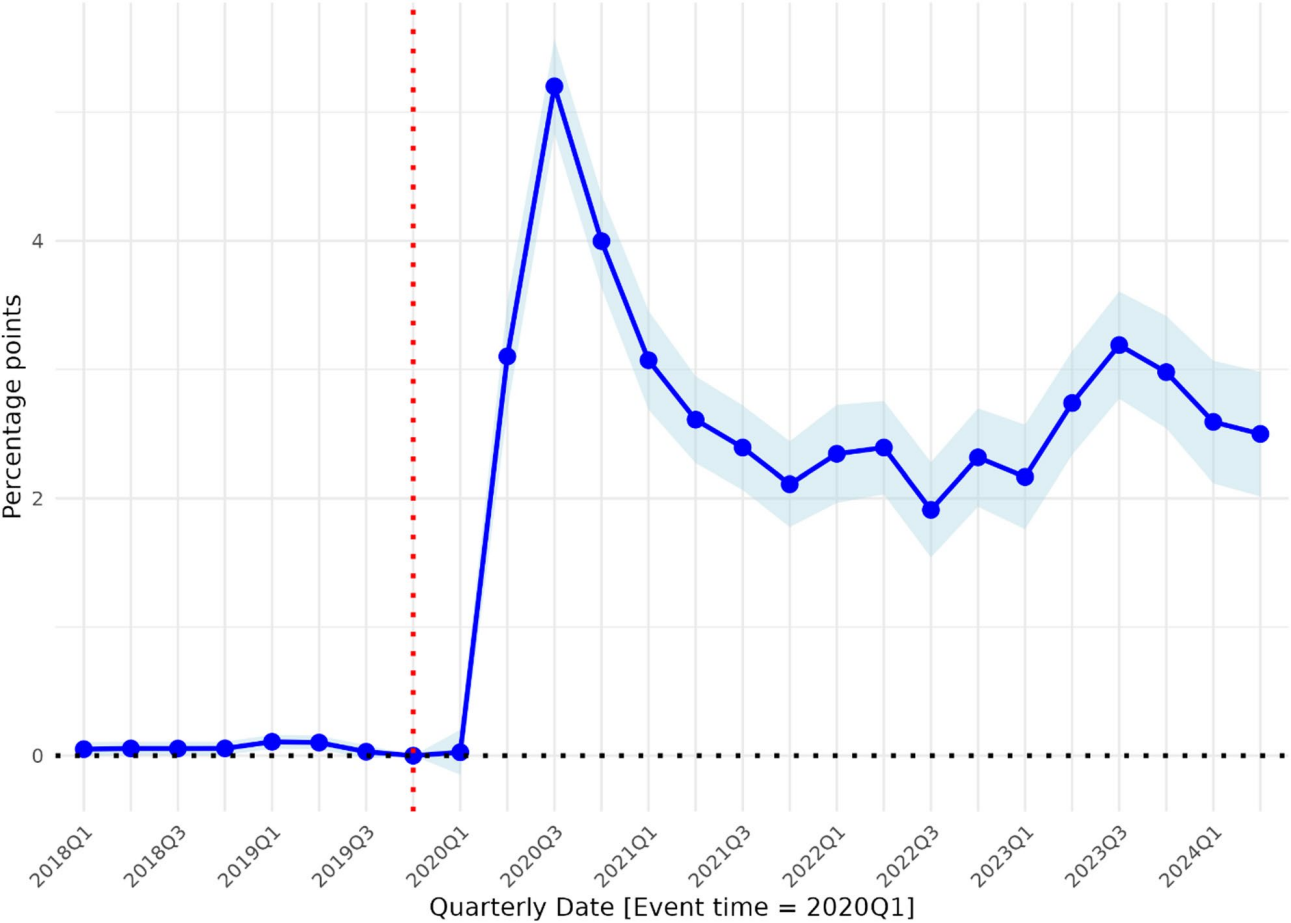
Event study figure for difference in the association of the 2020 Medicare Telehealth Policy Expansion on the use of telehealth between MA enrollees with and without ADRD. Notes: The figure presents event-study estimates from pooled ordinary least squares linear probability models of quarterly telehealth use at the enrollee-quarter level. Coefficients represent differences in the probability of any telehealth use, expressed in percentage points, for beneficiaries with ADRD relative to those without ADRD, by event time. Event time is defined relative to 2020Q1 (event time = 0), with 2019Q4 serving as the omitted reference period. The estimates show a sharp increase in telehealth use among beneficiaries with ADRD relative to those without ADRD beginning in 2020 quarter 1, followed by a decline from the early pandemic peak, with differences remaining positive in subsequent quarters. Models control for baseline demographic and comorbid conditions. Points denote coefficient estimates and shaded areas indicate 95% confidence intervals based on standard errors clustered at the enrollee (patient) level. All estimates are reported in percentage points

Figure 3 presents the main event study analysis of telehealth use among non-ADRD and ADRD MA enrollees, showing differential changes by quarter from 2018 to 2024. The corresponding coefficients are reported in Table A1. Whereas the DiD shows only pre-post comparisons, the event study shows the pattern by quarter. The event-study estimates show no evidence of differential pre-trends in telehealth use between the two groups prior to 2020. Although some of the pre-2020 coefficients are not statistically significant at conventional levels, the estimates are small in magnitude, centered near zero, and do not exhibit a systematic upward or downward pattern. In contrast, a sharp and sustained increase in telehealth use is observed immediately following the 2020Q1 policy expansion, supporting the plausibility of the parallel trends assumption – in the absence of the pandemic, there is reason to expect that telehealth use among individuals with and without ADRD would follow similar trajectories over time. Following the telehealth policy expansion in late Q1 2020, telehealth use among MA enrollees with ADRD rose sharply, peaking in Q3 2020 with 5.2 percentage points (95% CI, 4.83, 5.57) increase relative to non-ADRD enrollees. Although the gap narrowed, it remained positive and statistically significant through Q2 2024, indicating a sustained difference in use.

## Robustness checks

We conducted three robustness analyses to assess the sensitivity of our main findings to sample definition and outcome construction. The results are given in Table A2 and Figures A2 – A4. First, to address potential bias from attrition over the extended follow-up period, we restricted the sample to beneficiary-quarters observed between 2018 and 2022. Under this restriction, the estimated post-policy differential increase in telehealth use among MA beneficiaries with ADRD remained positive and of similar magnitude (ADRD × Post = 2.42 percentage points (95% CI, 2.23 to 2.62) compared with the main analysis (2.58 percentage points). Second, to assess whether results were driven by changes in the mix of services delivered via telehealth, we limited the analysis to office and other outpatient evaluation and management (E/M) claims, which represent a standard focus in empirical analyses of telehealth use. E/M related telehealth use yielded a larger estimated differential increase (4.74 percentage points; 95% CI, 4.52 to 4.96), indicating that the relative growth in telehealth use among beneficiaries with ADRD was particularly pronounced for E/M services. Third, we re-estimated all models including all enrolled beneficiary-quarters (including quarters with no medical claims). When patient-quarters with no medical claims were retained, rather than excluded as in the main analysis, the estimated differential increase was attenuated but remained statistically significant (0.74 percentage points; 95% CI, 0.61 to 0.86), consistent with mechanical dilution of telehealth use when non-utilization quarters are included. Taken together, these analyses indicate that the main findings are robust to alternative sample restrictions and outcome definitions, with differences in magnitude aligning with the underlying population and utilization mechanisms captured in each specification.

## Discussion

Although telehealth use has increased substantially since Q2 2020 when Medicare expanded the requirements to participate, long-term utilization among those with ADRD remains underexplored. This is a critical gap given the unique access challenges faced by individuals with ADRD, such as cognitive impairment, mobility limitations, and the need for caregiver assistance. What little research exists has been limited to FFS Medicare and in earlier years or with very small samples- leaving limited understanding of how telehealth is used in MA [9–11], which now enrolls over half of all Medicare beneficiaries [13].

In this study, we use Optum’s de-identified Clinformatics^®^ Data Mart Database (2017–2024) to examine telehealth use in MA beneficiaries living with and without ADRD. Traditionally, MA enrollees have had greater access to telehealth services compared to those in FFS Medicare [26], and one would expect providers and beneficiaries to be more likely to utilize telehealth, even during the pre-pandemic timeframe. We construct two longitudinal cohorts: those with an ADRD diagnosis prior to 2018 and those without any ADRD diagnosis during the study period. Our findings indicate that both cohorts saw a sharp rise in telehealth use in 2020, peaking mid-year, then declining and stabilizing by 2021. Throughout, individuals with ADRD consistently demonstrated modestly higher levels of use, but this modestly higher telehealth use may reflect greater underlying care needs and more frequent clinical contact among beneficiaries with ADRD, rather than improved access.

Several factors may plausibly contribute to the slightly higher telehealth use observed among beneficiaries with ADRD. First, people living with ADRD often have high clinical complexity and may require more frequent follow-up, medication management, and support for behavioral and psychological symptoms; telehealth may provide a practical complement to in-person care for some of these needs [9, 10]. Second, telehealth can reduce transportation and mobility barriers that are common among individuals with cognitive and functional impairment [9]. Third, caregiver availability and support may facilitate telehealth access for beneficiaries with ADRD by assisting with technology, scheduling, and communication during visits [11].

However, our findings also highlight an important and under-discussed trend: despite continued policy support for telehealth, utilization declined significantly after the peak of the pandemic. Prior analyses of Medicare telehealth similarly document rapid pandemic-era adoption followed by partial reversion as in-person care resumed [15, 17, 20]. This suggests that the initial spike in telehealth use may have been driven more by urgency and fear than by lasting changes in care delivery preferences or infrastructure. The modest policy effects we observe – compared to the steep pandemic-driven increase – raise questions about how to build and sustain meaningful engagement with telehealth once the immediate crisis has passed. Simply maintaining regulatory flexibility may not be enough; additional investment in technology access, digital literacy, provider incentives, and caregiver integration may be necessary to ensure telehealth remains a viable and effective option for high-need populations.

Finally, sustained telehealth use may be particularly valuable for people living with ADRD because it can reduce caregiver burden and support care continuity. Caregivers frequently facilitate telehealth visits by managing technology, ensuring attendance, and supporting communication—functions that may be harder to fulfill for in-person visits that require travel and time off work. Evidence from telehealth interventions in dementia caregiving suggests potential benefits for caregivers, including reductions in caregiver burden and related distress, and trials of caregiver-focused telehealth programs have demonstrated improvements in caregiver outcomes. Telehealth may also support continuity by enabling more frequent follow-up and allowing caregivers to join visits remotely, which may improve information exchange and care planning. Future research should examine how caregiver involvement shapes telehealth use and outcomes for ADRD dyads and what policy or practice supports best facilitate sustained, high-quality virtual care.

### Limitations

This study has several limitations. First, our study is observational. Although the matched DiD and event-study designs help account for time-invariant differences and common temporal shocks, residual confounding may remain; accordingly, our estimates should be interpreted as differential changes in telehealth use for beneficiaries with ADRD relative to those without, rather than the overall causal effect of the 2020 telehealth expansion. As with any DiD design, our results also rely on the assumption that pre-2020 trends in the ADRD–non-ADRD gap would have been parallel in the absence of the 2020 expansion and the pandemic; the pre-period event-study estimates are consistent with (but do not definitively confirm) this assumption. Second, the study is restricted to MA enrollees in one database (Optum Clinformatics), albeit one that contains approximately a quarter of all MA enrollees [27]; analyses using the full MA census and fee-for-service Medicare would help assess generalizability. Third, to ensure longitudinal consistency, we restricted the sample to individuals continuously enrolled and alive in 2017–2018 and excluded those newly diagnosed with ADRD after 2017, which may bias the cohort toward healthier individuals, particularly given average survival after ADRD diagnosis of 3–12 years [28]. Fourth, our claims data do not reliably capture care setting (e.g., long-term nursing home residence) over time. Because individuals with ADRD are more likely to receive institutional care, differences in care setting and facility-based practice patterns may contribute to observed differences in telehealth use. Fifth, we measure only whether a beneficiary had any telehealth use in a quarter and cannot assess visit frequency, modality (video vs. audio-only), or clinical appropriateness. Sixth, early 2020 telehealth use may be undercounted due to billing code overlap when payers allowed telehealth visits to be billed using standard in-person codes. Seventh, ADRD diagnoses in claims data may be subject to underreporting or misclassification [29, 30], which could bias ADRD classification. Finally, we do not examine downstream clinical outcomes or caregiver impacts—important areas for future research. Despite these limitations, following large, real-world MA cohorts over an extended period provides valuable evidence on telehealth use patterns among medically complex populations.

## Conclusion

In summary, telehealth use rose sharply among Medicare Advantage beneficiaries during the COVID-19 public health emergency and contemporaneous telehealth policy expansions, with beneficiaries living with ADRD experiencing a modest, largely temporary relative increase compared with matched beneficiaries without ADRD. Telehealth use declined after the early pandemic peak and the ADRD–non-ADRD difference narrowed over time, suggesting that the circumstances that drove rapid pandemic-era adoption did not translate into sustained higher uptake even among populations with substantial access barriers. Longer-term use may be constrained by ongoing obstacles such as technology access, digital literacy, and the complexity of coordinating care for individuals with cognitive impairment. These findings underscore the importance of moving beyond temporary policy flexibilities toward more durable approaches that support telehealth as an enduring, equitable mode of care, including caregiver-inclusive workflows, user-centered technology, and clinical models tailored to the needs of people living with ADRD. As Medicare telehealth policy continues to evolve, continued research should examine how to integrate telehealth into broader care strategies that are both effective and sustainable for high-need subgroups.

## Supplementary Information

Below is the link to the electronic supplementary material.

Supplementary Material 1

## Data Availability

The study uses proprietary, de-identified claims data licensed from Optum’s Clinformatics^®^ Data Mart and subject to a data use agreement. As such, the dataset of this study are not publicly available and cannot be shared due to confidentiality.
