# Supplementary material for: Telehealth among Medicare Advantage beneficiaries with and without Alzheimer's Disease and Related Dementias (ADRD), 2018–2024: from pandemic-era surge to post-pandemic decline
Source: BMC Health Serv Res. 2026 Mar 13;26:560. doi: 10.1186/s12913-026-14174-1 (PMC13097736; doi:10.1186/s12913-026-14174-1)
Supplement: Supplementary file 1 — Supplementary Material 1 [file 12913_2026_14174_MOESM1_ESM.docx]

**Additional File 1**

**Contents**

[Supplemental Methods 2](#_Toc219816751)

[ADRD ICD 10 codes 3](#_Toc219816752)

[Table A1: Event Study Estimation: Telehealth Use Among MA Beneficiaries With and Without ADRD, After and Before the 2020 Medicare Telehealth Policy Expansion 4](#_Toc219816753)

[Table A2: Difference-in-Differences Estimates of Telehealth Use Between MA Beneficiaries With and Without ADRD Before and After the 2020 Medicare Telehealth Policy Expansion 5](#_Toc219816754)

[Figure A1: Covariate Balance Before and After Propensity Score Matching 6](#_Toc219816755)

[Figure A2: Event study figure for difference in the association of the 2020 Medicare Telehealth Policy Expansion on the use of telehealth between MA enrollees with and without ADRD: Sample restricted to 2018 - 2022 7](#_Toc219816756)

[Figure A3: Event study figure for difference in the association of the 2020 Medicare Telehealth Policy Expansion on the use of telehealth between MA enrollees with and without ADRD: Sample restricted to E/M claims 8](#_Toc219816757)

[Figure A4: Event study figure for difference in the association of the 2020 Medicare Telehealth Policy Expansion on the use of telehealth between MA enrollees with and without ADRD: Sample includes all beneficiary-quarters with enrollment regardless of whether the beneficiary had any medical claim 9](#_Toc219816758)

**Supplemental Methods**

To further strengthen internal validity, we implemented propensity score matching prior to difference-in-differences estimation. Propensity scores were estimated using demographic and comorbidity characteristics measured in 2017, and matching was performed using 1:1 nearest-neighbor matching without replacement with a caliper of 0.2. Covariate balance before and after matching was assessed using standardized differences.

Using the matched sample, we estimated the following difference-in-differences model at the enrollee-quarter level:

$Y_{i,q}=\beta_{0} + \beta_{1}{ADRD}_{i}+ \beta_{2}{Post}_{q}+\beta_{3}({ADRD}_{i}*{Post}_{q})+ \theta X_{i}+ \varepsilon_{iq}$

where $Y_{i,q}$ is an indicator for any telehealth use by beneficiary $i$in quarter $q$, with quarters spanning 2018 quarter 1 through 2024 quarter 2. ${ADRD}_{i}$ indicates whether the beneficiary had an ADRD diagnosis in 2017, and ${Post}_{q}$ is an indicator for quarters beginning in 2020 quarter 1, following the Medicare telehealth policy expansion. $X_{i}$ includes baseline demographic and comorbidity covariates. Standard errors were clustered at the enrollee level. Beneficiary-quarters with no medical claims were excluded from the analysis.

The coefficient $\beta_{1}$captures baseline differences in telehealth use between beneficiaries with and without ADRD, while the coefficient of interest, $\beta_{3}$, represents the difference-in-differences estimator identifying changes in telehealth use for beneficiaries with ADRD beyond baseline differences following the policy expansion.

We also estimated an event-study specification to assess the plausibility of the parallel trends assumption:

${}$ $Y_{i,q} = \beta_{0} + \beta_{1}{ADRD}_{i} + \sum\sigma_{q}\left( Time_{q} \right) +\sum\mu_{q}\left( {ADRD}_{i}\cdot Time_{q} \right) +\theta X_{i}+ \varepsilon_{iq}$

where $Time_{q}$is an indicator for each quarter. The coefficients $\mu_{q}$capture quarter-specific differences in telehealth use between beneficiaries with and without ADRD, allowing for visual and statistical assessment of pre-expansion trends and dynamic treatment effects over time.

**ADRD ICD 10 codes**

The following ICD 10 codes were used to identify ADRD:

- Vascular dementia: F01.50-F01.51
- Dementia: F02.80-F02.81
- Unspecified dementia: F03.90-F03.91,
- Amnestic disorder: F04
- Alzheimer’s disease: G30.0/30.1/30.8/G30.9
- Pick’s disease: G31.01
- frontotemporal dementia: G31.09
- dementia with Lewy bodies: G31.83
- senile degeneration: G31.1
- Degeneration of nervous system: G31.2
- Age-related cognitive decline: R41.8

**Table A1**: Event Study Estimation: Telehealth Use Among MA Beneficiaries With and Without ADRD, After and Before the 2020 Medicare Telehealth Policy Expansion

| **Quarters** | **Even study coefficients**  **(Estimate, 95% CI)** |
| --- | --- |
| 2018Q1 | 0.05 [-0.01, 0.11] |
| 2018Q2 | 0.06 [ 0.00, 0.11] |
| 2018Q3 | 0.06 [ 0.00, 0.11] |
| 2018Q4 | 0.06 [ 0.00, 0.11] |
| 2019Q1 | 0.11 [ 0.05, 0.17] |
| 2019Q2 | 0.10 [ 0.04, 0.16] |
| 2019Q3 | 0.03 [-0.04, 0.10] |
| 2020Q1 | 0.03 [-0.15, 0.21] |
| 2020Q2 | 3.10 [ 2.68, 3.52] |
| 2020Q3 | 5.20 [ 4.83, 5.57] |
| 2020Q4 | 4.00 [ 3.64, 4.36] |
| 2021Q1 | 3.07 [ 2.69, 3.46] |
| 2021Q2 | 2.61 [ 2.27, 2.95] |
| 2021Q3 | 2.39 [ 2.06, 2.72] |
| 2021Q4 | 2.11 [ 1.78, 2.44] |
| 2022Q1 | 2.34 [ 1.96, 2.72] |
| 2022Q2 | 2.39 [ 2.03, 2.76] |
| 2022Q3 | 1.91 [ 1.54, 2.28] |
| 2022Q4 | 2.32 [ 1.94, 2.70] |
| 2023Q1 | 2.17 [ 1.76, 2.57] |
| 2023Q2 | 2.74 [ 2.34, 3.14] |
| 2023Q3 | 3.19 [ 2.77, 3.61] |
| 2023Q4 | 2.98 [ 2.54, 3.42] |
| 2024Q1 | 2.59 [ 2.12, 3.07] |
| 2024Q2 | 2.50 [ 2.02, 2.98] |
|  |  |
| Enrollee-quarter (N) | 4,837,037 |
| Unique enrollees | 421,131 |

**Notes**: The table reports event-time interaction coefficients from pooled ordinary least squares linear probability models estimated using an event-study difference-in-differences design. Event time is defined relative to 2020Q1. Coefficients represent differences in the probability of any telehealth use, expressed in percentage points, for beneficiaries with ADRD relative to those without ADRD, by event time. Estimates are normalized to 2019Q4, which serves as the omitted pre-event reference period.

**Table A2**: Difference-in-Differences Estimates of Telehealth Use Between MA Beneficiaries With and Without ADRD Before and After the 2020 Medicare Telehealth Policy Expansion

|  | Sample restricted to 2018 - 2022 | Sample restricted to E/M claims | Includes All patient quarters |
| --- | --- | --- | --- |
|  | **(Estimate, 95% CI)** | **(Estimate, 95% CI)** | **(Estimate, 95% CI)** |
| Constant | 15.26 [14.70, 15.82] | 12.36 [11.69, 13.04] | 14.46 [13.95, 14.97] |
| ADRD | 0.34 [ 0.28, 0.40] | 0.49 [ 0.43, 0.55] | 0.21 [ 0.16, 0.26] |
| Post | 5.87 [ 5.76, 5.98] | 3.98 [ 3.88, 4.08] | 3.19 [ 3.12, 3.27] |
| ADRD x Post | 2.42 [ 2.23, 2.62] | 4.74 [ 4.52, 4.96] | 0.74 [ 0.61, 0.86] |
| Demographics |  |  |  |
| Age | -0.16 [-0.17, -0.16] | -0.13 [-0.14, -0.12] | -0.17 [-0.17, -0.16] |
| Female | 0.58 [ 0.50, 0.67] | 0.64 [ 0.54, 0.73] | 0.59 [ 0.52, 0.66] |
| Dual Eligibility | 0.38 [ 0.30, 0.47] | 0.43 [ 0.32, 0.54] | 0.38 [ 0.30, 0.45] |
| Rural Residence | -0.52 [-0.70, -0.34] | -1.64 [-1.83, -1.46] | 0.08 [-0.09, 0.25] |
| Comorbidities |  |  |  |
| Asthma | 1.22 [ 1.05, 1.39] | 0.95 [ 0.76, 1.14] | 1.31 [ 1.15, 1.46] |
| COPD | 0.12 [ 0.01, 0.22] | 0.13 [ 0.01, 0.26] | 0.01 [-0.09, 0.10] |
| Diabetes | 0.44 [ 0.35, 0.52] | 0.36 [ 0.26, 0.45] | 0.56 [ 0.49, 0.63] |
| Hypertension | 0.47 [ 0.36, 0.57] | 0.19 [ 0.07, 0.32] | 0.88 [ 0.80, 0.96] |
| Cancer | 0.47 [ 0.37, 0.58] | 0.23 [ 0.11, 0.35] | 0.61 [ 0.52, 0.70] |
| Arthritis | 0.69 [ 0.61, 0.77] | 0.43 [ 0.33, 0.52] | 0.90 [ 0.83, 0.97] |
| Congestive heart failure | 0.19 [ 0.09, 0.30] | 0.14 [ 0.02, 0.26] | -0.09 [-0.19, 0.00] |
| Coronary artery disease | 0.46 [ 0.37, 0.55] | 0.33 [ 0.23, 0.44] | 0.62 [ 0.54, 0.70] |
| Stroke | 0.06 [-0.06, 0.18] | 0.06 [-0.07, 0.20] | -0.11 [-0.21, 0.00] |
| Kidney disease | 0.14 [ 0.05, 0.22] | 0.05 [-0.05, 0.15] | -0.20 [-0.28, -0.13] |
| Liver disease | 0.45 [ 0.26, 0.64] | 0.38 [ 0.16, 0.60] | 0.26 [ 0.08, 0.43] |
| Depression | 1.21 [ 1.12, 1.29] | 1.35 [ 1.25, 1.45] | 0.99 [ 0.92, 1.07] |
| Enrollee-quarter (N) | 4,331,493 | 3,672,944 | 6,262,093 |
| Unique enrollees | 420,921 | 370,047 | 445,270 |

**Notes:** Estimates are from pooled OLS linear probability model estimated using a difference-in-differences (DID) design. The unit of analysis is the enrollee-quarter, and the outcome is an indicator for any telehealth use during the quarter. *Post* indicates the post-2020 period. ADRD indicates whether the enrollee was diagnosed with Alzheimer’s disease or related dementias. The interaction term (*ADRD × Post*) represents the DID estimate, capturing the differential change in telehealth use among enrollees with ADRD in the post period relative to those without ADRD. Standard errors are clustered at the enrollee (patient) level. All coefficients and 95% confidence intervals are reported in percentage points. The adjusted model additionally controls for **baseline** age (years), sex, comorbid conditions (asthma, COPD, diabetes, hypertension, cancer, arthritis, congestive heart failure, coronary artery disease, stroke, kidney disease, liver disease, depression), dual eligibility status, and rural residence.

**Figure A1**: Covariate Balance Before and After Propensity Score Matching


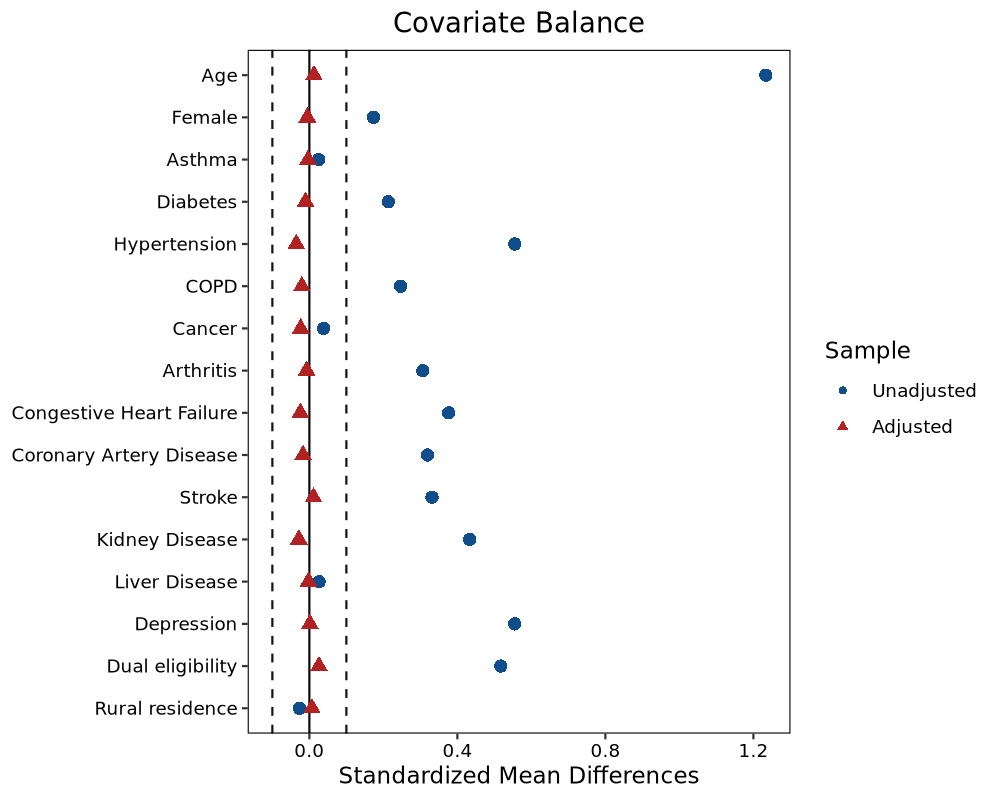


Notes: The figure displays standardized mean differences for baseline covariates before and after matching. Points to the left (right) of zero indicate lower (higher) values among beneficiaries with ADRD relative to those without ADRD. The vertical reference line at 0.10 denotes the conventional threshold for acceptable covariate balance. Covariates are measured at baseline.

**Figure A2**: Event study figure for difference in the association of the 2020 Medicare Telehealth Policy Expansion on the use of telehealth between MA enrollees with and without ADRD: Sample restricted to 2018 - 2022


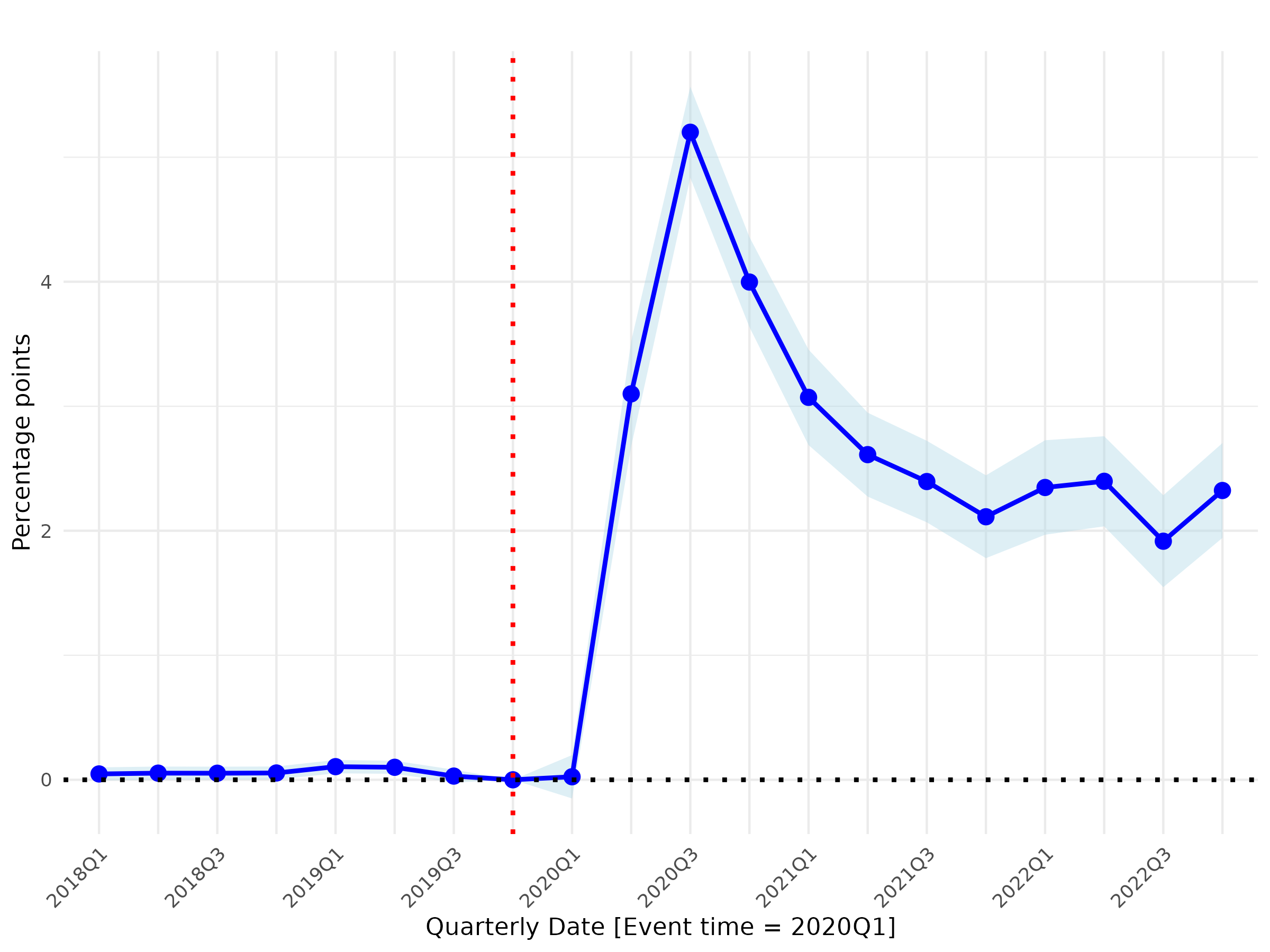


**Notes:** The figure presents event-study estimates from pooled ordinary least squares linear probability models of quarterly telehealth use at the enrollee-quarter level for a sample restricted to 2018 – 2022. Coefficients represent differences in the probability of any telehealth use, expressed in percentage points, for beneficiaries with ADRD relative to those without ADRD, by event time. Event time is defined relative to 2020Q1 (event time = 0), with 2019Q4 serving as the omitted reference period. Models control for baseline demographic and comorbid conditions. Points denote coefficient estimates and shaded areas indicate 95% confidence intervals based on standard errors clustered at the enrollee (patient) level. All estimates are reported in percentage points.

**Figure A3**: Event study figure for difference in the association of the 2020 Medicare Telehealth Policy Expansion on the use of telehealth between MA enrollees with and without ADRD: Sample restricted to E/M claims


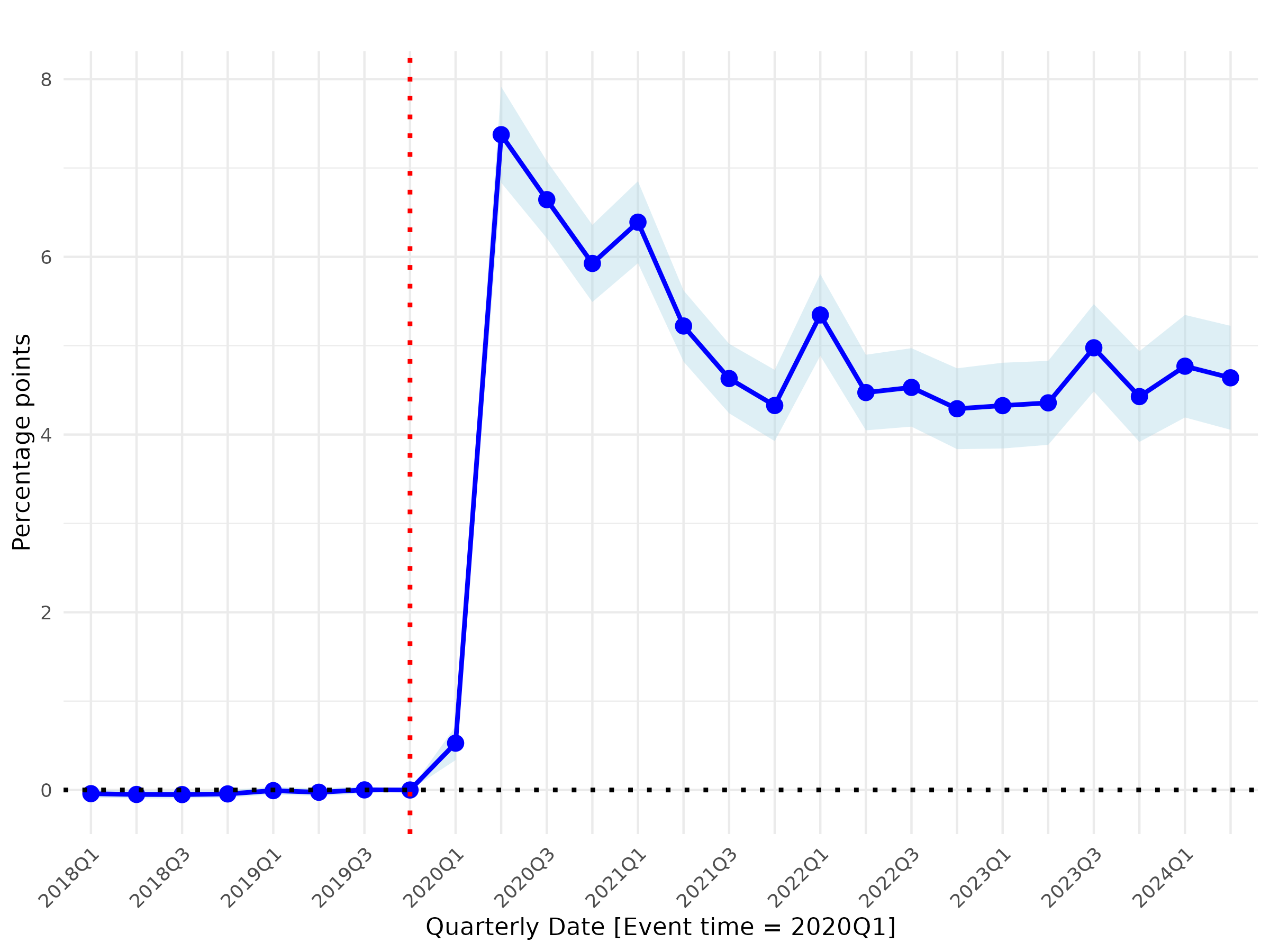


**Notes:** The figure presents event-study estimates from pooled ordinary least squares linear probability models of quarterly telehealth use at the enrollee-quarter level. The outcome is any E/M telehealth use in a quarter for a sample restricted to E/M claims. Coefficients represent differences in the probability of any telehealth use, expressed in percentage points, for beneficiaries with ADRD relative to those without ADRD, by event time. Event time is defined relative to 2020Q1 (event time = 0), with 2019Q4 serving as the omitted reference period. Models control for baseline demographic and comorbid conditions. Points denote coefficient estimates and shaded areas indicate 95% confidence intervals based on standard errors clustered at the enrollee (patient) level. All estimates are reported in percentage points.

**Figure A4**: Event study figure for difference in the association of the 2020 Medicare Telehealth Policy Expansion on the use of telehealth between MA enrollees with and without ADRD: Sample includes all beneficiary-quarters with enrollment regardless of whether the beneficiary had any medical claim


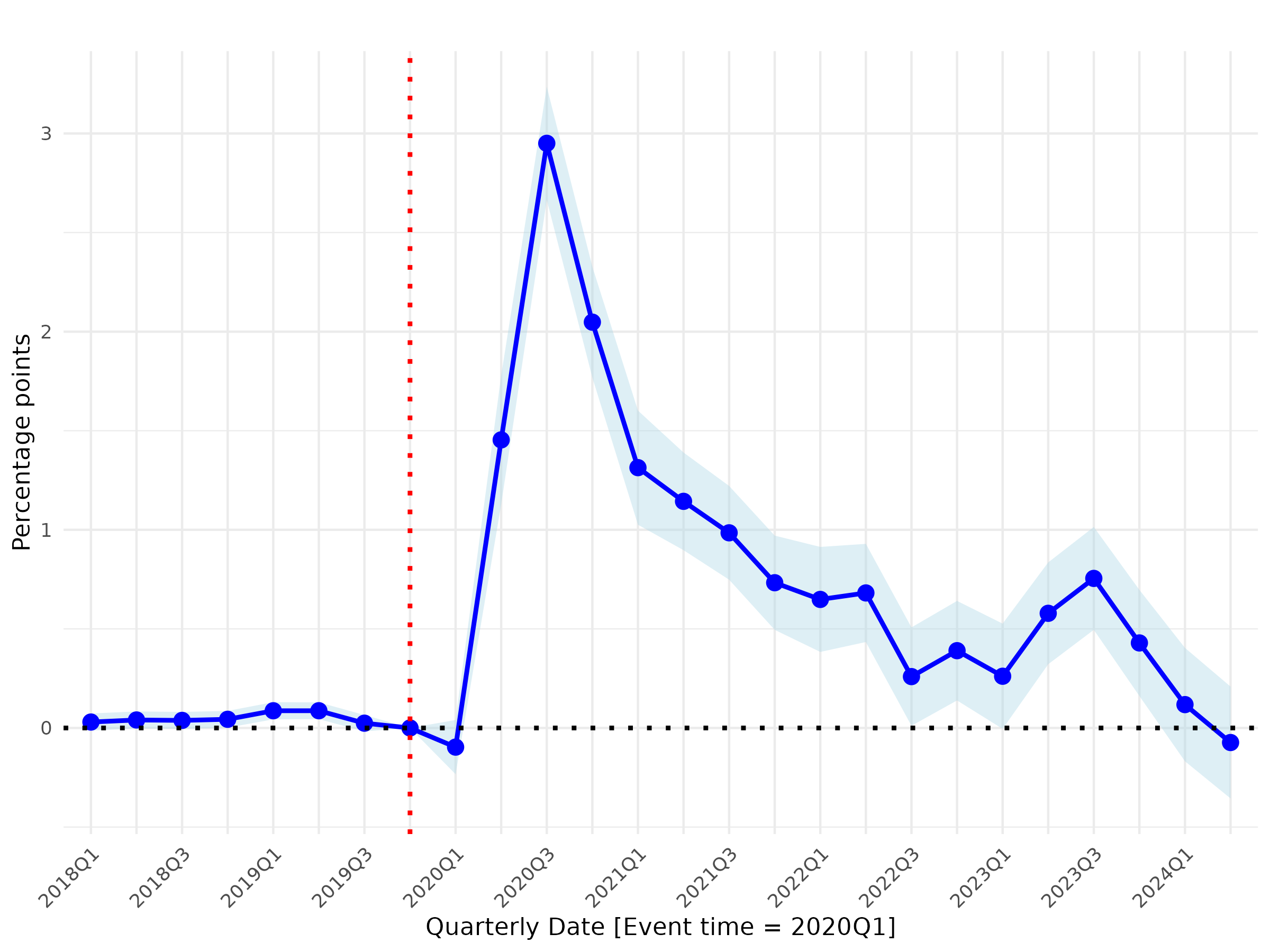
**Notes:** The figure presents event-study estimates from pooled ordinary least squares linear probability models of quarterly telehealth use at the enrollee-quarter level. The result is for the sample that includes all quarters for which a beneficiary is enrolled regardless of whether they use any medical care or not for the quarter. Coefficients represent differences in the probability of any telehealth use, expressed in percentage points, for beneficiaries with ADRD relative to those without ADRD, by event time. Event time is defined relative to 2020Q1 (event time = 0), with 2019Q4 serving as the omitted reference period. Models control for baseline demographic and comorbid conditions. Points denote coefficient estimates and shaded areas indicate 95% confidence intervals based on standard errors clustered at the enrollee (patient) level. All estimates are reported in percentage points.
